# Expression of concern: High performance flexible supercapacitors based on secondary doped PEDOT–PSS–graphene nanocomposite films for large area solid state devices

**DOI:** 10.1039/d4ra90075h

**Published:** 2024-07-08

**Authors:** Syed Khasim, Apsar Pasha, Nacer Badi, Mohana Lakshmi, Yogendra Kumar Mishra

**Affiliations:** a Department of Physics, Faculty of Science, University of Tabuk Tabuk Kingdom of Saudi Arabia syed.pes@gmail.com; b Renewable Energy Laboratory, Nanotechnology Research Unit, Faculty of Science, University of Tabuk Tabuk Kingdom of Saudi Arabia; c Department of Physics, PES University Bangalore 560100 Karnataka India; d Department of Physics, Gousia College of Engineering Ramanagaram Karnataka India; e Mads Clausen Institute, Nano SYD, University of Southern Denmark Alsion 2 Sønderborg Denmark

## Abstract

Expression of concern for ‘High performance flexible supercapacitors based on secondary doped PEDOT–PSS–graphene nanocomposite films for large area solid state devices’ by Syed Khasim *et al.*, *RSC Adv.*, 2020, **10**, 10526–10539, https://doi.org/10.1039/D0RA01116A.

*RSC Advances* is publishing this expression of concern in order to alert our readers that we are presently unsure of the reliability of the data reported in Fig. 7 of this article. In addition, two images presented in Fig. 2 appear to have been reproduced from previous publications by different authors without appropriate acknowledgement.

An investigation is underway, and an expression of concern will continue to be associated with the article until a final outcome is reached.

Laura Fisher

3^rd^ July 2024

Executive Editor, *RSC Advances*

